# High glucose induces pyroptosis of retinal microglia through NLPR3 inflammasome signaling

**DOI:** 10.5935/0004-2749.20210010

**Published:** 2025-02-02

**Authors:** Libin Huang, Junmei You, Yao Yao, Maosong Xie

**Affiliations:** 1 The First Affiliated Hospital of Fujian Medical University, China

**Keywords:** Diabetic retinopathy, Microglia, NLRP3 inflammasome, Pyroptosis, Gasdermin D, Retinopatia diabética, Microglia, NLRP3 Inflammassomos, Piroptose, Gasdermin D

## Abstract

**Purpose:**

Diabetic retinopathy is currently considered a chronic inflammatory disease involving NOD-like receptor family pyrin domain containing 3 inflammasome activation and retinal microglial pyroptosis. In this study, we aimed to investigate whether NOD-like receptor family pyrin domain containing 3 inflammasome signaling induces pyroptotic death of retinal microglia under high-glucose conditions.

**Methods:**

Retinal microglia were stimulated by high glucose levels for 24 h. Cell viability, lactate dehydrogenase release, and caspase-1 activity were detected *in vitro*. The expression of pro-inflammatory cytokine (interleukin-1β, activated microglia marker ionized calcium-binding adapter molecule-1), NOD-like receptor family pyrin domain containing 3, cleaved caspase-1, and cleaved gasdermin D were examined. Subsequently, retinal microglia were pretreated with the inhibitors of NOD-like receptor family pyrin domain containing 3 inflammasome signaling prior to stimulation with high glucose, and their molecular and functional changes were evaluated.

**Results:**

High-glucose (25, 50, or 100 mM) stimulation decreased cell viability, but enhanced lactate dehydrogenase release and caspase-1 activity in a dose-dependent manner. Moreover, high glucose upregulated the protein expression of interleukin-1β, ionized calcium-binding adapter molecule-1, NOD-like receptor family pyrin domain containing 3, cleaved caspase-1, and cleaved gasdermin D. However, pretreatment with the inhibitors of NOD-like receptor family pyrin domain containing 3 inflammasome signaling inhibited high glucose (25 mM)-induced cytotoxicity, NOD-like receptor family pyrin domain containing 3 inflammasome activation, and pyroptosis of retinal microglia.

**Conclusions:**

NOD-like receptor family pyrin domain containing 3 inflammasome signaling may modulate retinal microglia-related inflammation and pyroptosis under high-glucose conditions.

## Abstract (pt)

**Objetivo:**

Atualmente, a retinopatia diabética é considerada uma doença inflamatória crônica envolvendo a ativação de inflamassomas NLRP3 e piroptose da micróglia da retina. Neste estudo, objetivamos investigar se a sinalização de inflamassomas NLRP3 induz a morte da micróglia da retina sob condições de alta glicose.

**Métodos:**

A micróglia da retina foi estimulada por altos níveis de glicose durante 24 horas. A viabilidade celular, a liberação de LDH e a atividade da caspase1 foram analisadas *in vitro*. Avaliou-se a expressão de citocina pró-inflamatória (IL1β), de marcador de micróglia ativado (Iba1), de NLRP3, de caspase1 clivada e de GSDMD clivada. Subsequentemente, a micróglia da retina foi pré-tratada com inibidores da sinalização de inflamassomas NLRP3 antes da estimulação com altos níveis de glicose e suas alterações moleculares e funcionais foram avaliadas.

**Resultados:**

A estimulação com altos níveis de glicose (25 mM, 50 mM ou 100 mM) diminuiu a viabilidade celular, mas aumentou a liberação de LDH e a atividade da caspase1 de forma dependente da dose. Além disso, os altos níveis de glicose aumentaram a expressão das proteínas IL1β, Iba1, NLRP3, caspase1 clivada e GSDMD clivada. No entanto, o pré-tratamento com inibidores da sinalização de inflamassomas NLRP3 e a posterior estimulação com altos níveis de glicose (25 mM) induziu citotoxicidade, a ativação de inflamassomas NLRP3 e a piroptose da micróglia da retina.

**Conclusão:**

A sinalização de inflamassomas NLRP3 pode modular a inflamação e a piroptose da micróglia da retina na presença de altos níveis de glicose.

## INTRODUCTION

Diabetic retinopathy (DR), the major ocular complication of diabetes mellitus, is the leading cause of blindness in working-age populations^(1)^. Numerous studies have documented the effectiveness of routine DR screening and early treatment^(2-3)^, while current therapies are unable to reverse the vision loss in the advanced background or proliferative stage of DR.

The exact mechanisms of DR remain unclear. Although DR has been traditionally described as a microvascular disorder, recent evidence demonstrated that early DR is linked to retinal inflammation associated with microglia activation^(4-5)^. Over-activated microglia (M1 phenotype) induce the release of inflammatory factors, contribute to the development of neurovascular unit lesions, and eventually lead to irreversible retinal dysfunction^(6-7)^.

The NOD-like receptor family pyrin domain containing 3 (NLRP3) inflammasome, one of the key sensors in microglial plasma, plays a critical role in inflammation mediated by inflammatory cytokines, such as interleukin-1β (IL-1β), from microglia^(8)^. NLRP3 recognizes different endogenous and exogenous stimuli, and combines with the adaptor apoptosis-associated speck-like protein containing a caspase activation and recruitment domain (ASC) and pro-caspase-1. Accordingly, they are assembled to a multiprotein complex^(9)^. Activation of the NLPR3 inflammasome activates pro-caspase-1 cleavage, facilitates IL-1β secretion, and subsequently triggers a highly inflammatory type of programmed cell death termed pyroptosis^(10)^. Gasdermin D (GSDMD) is a membrane pore-forming protein involved in pyroptosis^(11)^. The NLPR3 inflammasome could cleave GSDMD to generate the N-terminal fragment of GSDMD, cause cell rupture, and increase the release of IL-1β^(12)^.

It has been reported that NLRP3 inflammasome signaling is involved in the pathogenesis of various eye diseases^(13-15)^. Some studies have demonstrated that caspase-1 activity and IL-1β production were significantly increased in microglia *in vitro* following exposure to hyperglycemic conditions^(16-17)^, suggesting that microglial pyroptosis is a crucial factor in the pathogenesis of DR. A recent study found that NLRP3 gene knockout downregulated the expression of caspase-1 and proinflammatory cytokines, and alleviated retinal ganglion cell death following optic nerve crush injury^(18)^. Moreover, high glucose could trigger pyroptosis of retinal microvascular endothelial cells via the NLRP3 inflammasome signaling pathway^(19-20)^. However, it remains poorly understood whether the activation of the NLRP3 inflammasome causes pyroptosis of retinal microglia in DR.

In the present study, we constructed an *in vitro* model to determine the role of NLRP3 inflammasome signaling in modulating retinal microglial pyroptosis under high-glucose conditions. Additionally, we intended to interpret the pathogenesis of DR in terms of microglia pyroptosis and related inflammation.

## METHODS

### Primary retinal microglia culture

All animal procedures were approved by the Animal Care and Use Committee of the First Affiliated Hospital of Fujian Medical University (Fuzhou, China) (Approval No. 2016-YK-163), and conformed to the Association for Research in Vision and Ophthalmology Statement on the Use of Animals in Ophthalmic and Vision Research.

The primary microglial culture was performed as previously described^(21)^. In brief, retinas of healthy newborn C57BL/6 mice (Shanghai SLAC Laboratory Animal Co. Ltd., Shanghai, China) were digested with 0.125% trypsin for 30 min at 37°C to generate a single-cell suspension. Subsequently, the cells were resuspended in Dulbecco’s modified Eagle’s medium/F-12 culture medium containing 10% fetal bovine serum, 1% microglia growth supplement (Sciencell, USA), 100 U/mL penicillin, and 100 µg/mL streptomycin, plated onto 75 cm^2^ culture flasks, and incubated at 37°C in a humidified atmosphere containing 5% CO_2_. The culture medium was changed at 24 h. After 2 weeks, mixed glial cells were purified by shaking at 200 rpm for 1 h. The supernatant containing microglia were harvested and used in the following experiments. The purity of microglia was determined through flow cytometry using fluorescein isothiocyanate-conjugated rabbit anti-CD11b and isotype immunoglobulin G2b control antibodies (Abcam, Cambridge, UK).

### Cell treatment

Microglia were incubated with D-glucose 5.5 (control), 25, 50, and 100 mM (Sigma-Aldrich, St. Louis, MO, USA) for 24 h. The cells were pretreated with the NLRP3 inhibitor MCC950 (10 µM) or caspase-1 inhibitor Z-Tyr- Val-Ala-Asp(OMe) fluoromethyl ketone (Z-YVAD-FMK; 10 µM) (all from Sigma-Aldrich) for 30 min prior to treatment with high glucose to suppress NLRP3 inflammasome signaling.

### Cell viability

Cell viability was detected using the Cell Counting Kit-8 (CCK-8; Beyotime), according to the instructions provided by the manufacturer. After the indicated treatments, CCK-8 solution (10 µL) was added to each well and treated for 2 h at 37°C. The optical density was measured at 450 and 690 nm.

### Cytotoxicity assay

The levels of lactate dehydrogenase (LDH) in supernatants were determined using the LDH Cytotoxicity Assay Kit (Beyotime) as previously described^(22)^. Cytotoxicity (%) was calculated as follows: 100 × (experimental LDH - spontaneous LDH)/(maximum LDH release - spon taneous LDH).

### Caspase-1 activity analysis

After the indicated treatments, microglia were disintegrated and centrifuged to obtain cell lysates. Caspase-1 activity was detected using the Caspase-1 Activity Assay Kit (Beyotime) according to the instructions provided by the manufacturer.

### Evaluation of IL-1_β_ secretion in the supernatants

The concentration of IL-1β in the culture supernatants was determined using enzyme-linked immunosorbent assay kits (R&D Systems, MN, USA), based on the instructions provided by the manufacturer.

### Western blotting analysis

Total protein was extracted from the cells, and the con centration was determined using a bicinchoninic acid kit (Pierce, Rockford, IL, USA). The proteins were separated by electrophoresis on a 10% sodium dodecyl sulfate-polyacrylamide gel and transferred to a high-quality polyvinylidene difluoride membrane. After blocking with 5% nonfat milk in tris-buffered saline with Tween 20 solution, the membranes were incubated with primary antibodies overnight at 4°C. The following antibodies were used: rabbit anti-Iba-1 (1:200; Abcam); rabbit anti-NLRP3 (1:500; Abcam); rabbit anti-cleaved caspase-1 (1:500; Cell Signaling Technology, Danvers, MA, USA); rabbit anti-cleaved GSDMD (1:500; Cell Signaling Technology); and rabbit anti-glyceraldehyde3- phosphate dehydrogenase (anti-GAPDH: 1:1,000; Santa Cruz Biotechnology, Santa Cruz, CA, USA). After washing, each blot was incubated with horseradish peroxidase-conjugated secondary antibody (goat anti-rabbit immunoglobulin G-horseradish peroxidase; Santa Cruz Biotechnology, Santa Cruz, CA, USA) for 1 h, and visualized with enhanced chemiluminescence. The quantitation of each band was performed using the Quantity One software 4.6 (Bio-Rad Laboratories, Hercules, CA, USA) using GAPDH as an internal control.

### Statistical analysis

Data are expressed as the mean ± standard deviation. Statistical analysis was performed using one-way analysis of variance with the Tukey-Kramer multiple comparison test (GraphPad Prism, version 5.01; GraphPad, La Jolla, CA, USA). Statistical significance was set at p*<*0.05. Error bars indicate standard deviation.

## RESULTS

### Characterization and identification of retinal microglia *in vitro*

**Figure 1:**
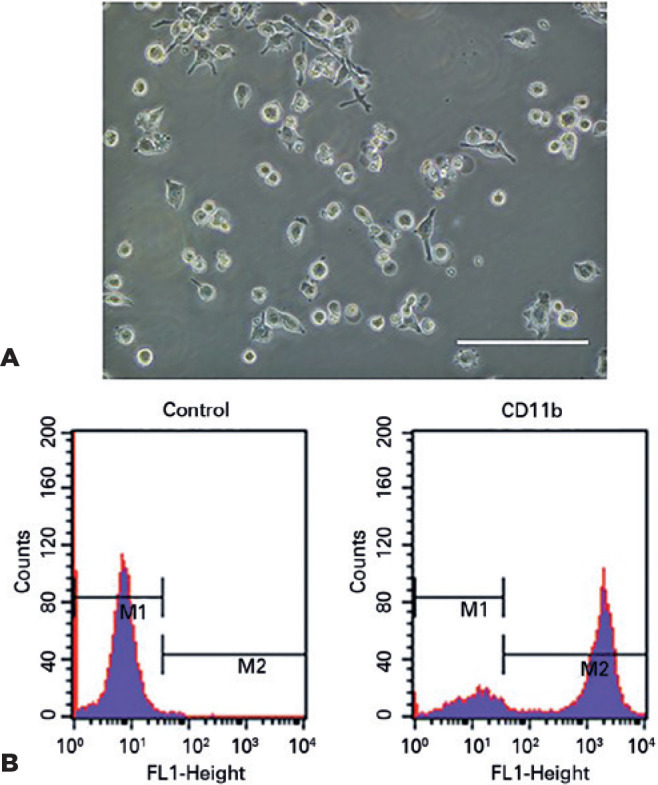
Morphology and identification of retinal microglia in culture. Primary retinal microglia appeared in either rounded, bipolar, or multipolar shapes (A). The surface expression rate of CD11b was 80.18 ± 4.38%. Six batches of microglia were analyzed using flow cytometry (B). Scale bars indicate 100 µm.

Primary retinal microglia showed either rounded, bipolar, or multipolar shapes (Figure 1A). The purity of retinal microglia was 80.18 ± 4.38%, determined through flow cytometry using a CD11b antibody (Figure 1B).

### High glucose affected the viability of retinal microglia *in vitro*

**Figure 2:**
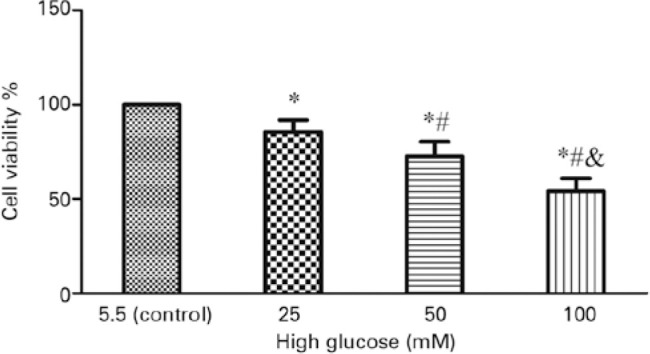
High glucose reduced the viability of retinal microglia *in vitro*. Retinal microglia were stimulated by different concentrations of high glucose (25, 50, and 100 mM). The cell viability was measured using the CCK-8 kit, which revealed a marked decline in a dose-dependent manner. Values are expressed as the mean ± SD. n=6.

As shown by the CCK-8 assay, incubation with high glucose (25, 50, or 100 mM) resulted in a remarkable decline in cell viability in a dose-dependent manner compared with the control (p<0.05) (Figure 2).

CCK-8= Cell Counting Kit-8; SD= standard deviation.

### High glucose activated retinal microglia *in vitro*

**Figure 3:**
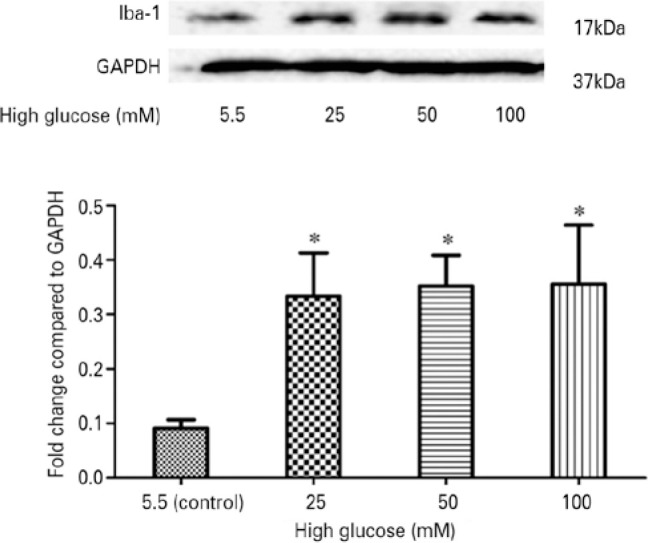
High glucose activated retinal microglia *in vitro*. Western blotting analysis indicated that treatment with high glucose upregulated the protein expression of Iba-1 in retinal microglia. There were no significant differences observed among the groups treated with different concentrations of high glucose (25, 50, and 100 mM).

Treatment with high glucose (25, 50, or 100 mM) upregulated the protein expression of Iba-1, which is a specific marker for activated microglia, compared with the control (p<0.05) (Figure 3). Western blotting analysis did not reveal significant differences among the groups treated with different concentrations of high glucose (p>0.05).

Values are expressed as the mean ± SD. n=3; ^*^p<0.05 versus the control group.

Iba-1= ionized calcium-binding adapter molecule-1; SD= standard deviation; GAPDH= glyceraldehyde-3-phosphate dehydrogenase.

### High glucose induced the activation of NLRP3 inflammasome signaling and pyroptosis of retinal microglia

**Figure 4:**
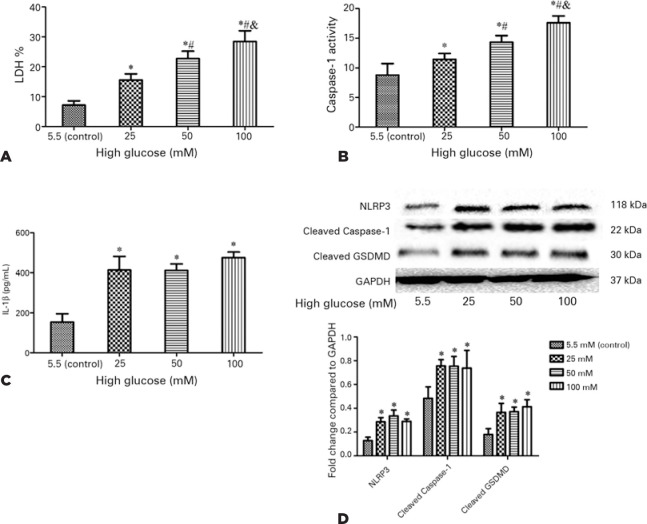
High glucose triggered NLRP3 inflammasome signaling and pyroptosis in retinal microglia. Treatment with high glucose (25, 50, and 100 mM) increased LDH release (A) and caspase-1 activity (B) in retinal microglia in a dose-dependent manner. Detection with ELISA showed that high glucose increased the release of IL-1_β_ (C). Moreover, western blotting analysis indicated that high glucose enhanced the protein expression of NLRP3, cleaved caspase-1, and cleaved GSDMD (D).

LDH release and caspase-1 activity in the high glucose-treated group (25, 50, or 100 mM) were higher than those measured in the control group (p<0.05) (Figure 4A, B), showing a dose-dependent effect.

Values are expressed as the mean ± SD. n=3; ^*^p<0.05 versus the control group; ^#^p<0.05 versus the 25 mM high glucose-treated group; ^&^p<0.05 versus the 50 mM high glucose-treated group.

NLRP3= NOD-like receptor family pyrin domain containing 3; LDH= lactate dehydrogenase; ELISA= enzyme-linked immunosorbent assay; IL-1β= interleukin-1β; GSDMD= gasdermin D; SD= standard deviation; GAPDH= glyceraldehyde-3-phosphate dehydrogenase.

The enzyme-linked immunosorbent assay showed that high glucose (25, 50, or 100 mM) significantly increased the secretion of IL-1β compared with the control (p<0.05) (Figure 4C). However, IL-1β secretion was not significantly different among the groups treated with different concentrations of high glucose (p>0.05).

Moreover, western blotting analysis indicated that high glucose upregulated the protein expression of NLRP3, cleaved caspase-1 and cleaved GSDMD in retinal microglia (p<0.05) (Figure 4D). There were no significant differences observed among the groups treated with different concentrations of high glucose (p>0.05).

### NLRP3 inflammasome signaling mediated high glucose-induced pyroptosis in retinal microglia

MCC950 or Z-YVAD-FMK were used to inhibit NLRP3 or caspase-1 for evaluating the effect of NLRP3 inflammasome signaling in high glucose-induced pyroptosis. As the degree of retinal microglia activation did not exhibit significant differences among the groups treated with different concentrations of high glucose, stimulation with the minimum concentration of high glucose (25 mM) was performed in subsequent experiments.

**Figure 5:**
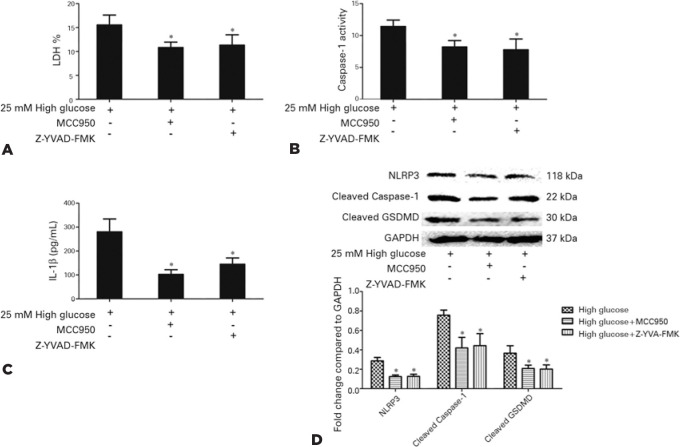
NLRP3 inflammasome signaling modulated high glucose-induced pyroptosis in retinal microglia. Retinal microglia were pretreated with the NLRP3 inhibitor MCC950 or caspase-1 inhibitor Z-YVAD-FMK prior to incubation with high glucose (25 mM). LDH release (A), caspase-1 activity (B), and IL-1_β_ secretion (C) by retinal microglia were inhibited, along with the decrease of the protein expression of NLRP3, cleaved caspase-1, and cleaved GSDMD (D).

As shown in figure 5, either MCC950 or Z-YVAD-FMK could suppress LDH release, caspase-1 activity, and IL-1β secretion in retinal microglia stimulated with high glucose (25 mM) (p<0.05). Additionally, western blotting analysis confirmed that the protein expression of NLRP3, cleaved caspase-1, and cleaved GSDMD was downregulated in retinal microglia (p<0.05).

Values are expressed as the mean ± SD. n=3; *p<0.05 versus the control group.

NLRP3= NOD-like receptor family pyrin domain containing 3; Z-YVAD-FMK= Z-Tyr-Val-Ala-Asp(OMe) fluoromethyl ketone; LDH, lactate dehydrogenase; IL-1β= interleukin-1β; GSDMD= gasdermin D; SD= standard deviation; GAPDH= glyceraldehyde3- phosphate dehydrogenase.

## DISCUSSION

The mechanisms triggering pyroptotic cell death during the development of DR are intricate and not fully understood^(23)^. In this study, we demonstrated that NLRP3 inflammasome signaling could modulate retinal microglial pyroptosis under high-glucose conditions and induce microglia-related inflammation in the retina.

As the crucial immunoregulatory cells in the retina, microglia play a pivotal role in the maintenance of ho meostasis in the retinal microvasculature, and are involved in various retinal diseases (especially DR)^(7,24)^. Metabolic abnormalities may initially give rise to microglial dysfunction^(25)^. The treatment of DR with mi croglia to alleviate retinal inflammation has been widely investigated^(26-27)^. Our results are in line with the current evidence^(28)^ that high glucose could decrease cell viability and activate retinal microglia *in vitro*. Iba-1, a calcium-binding protein, participates in the migration and phagocytosis of activated microglia^(29)^. The present study showed that there are no significant differences in Iba-1 expression among the groups treated with diffe rent concentrations of high glucose. We hypothesized that the migratory and phagocytic capability of activated retinal microglia may have already peaked after stimulation with 25 mM high glucose.

Upon excess activation, retinal microglia shifted to the M1 phenotype^(30)^, promoted the release of pro-inflammatory factors (e.g., IL-1β), and may subsequently result in the activation of pyroptosis to rupture microglia and further aggravate inflammatory responses in the retina^(31)^. We demonstrated that IL-1β secretion by retinal microglia was upregulated under high-glucose conditions, suggesting that hyperglycemia may induce phenotypic polarization of retinal microglia to generate proinflammatory effects *in vitro*. Moreover, high glucose increased LDH release and cleaved caspase-1/cleaved GSDMD expression, which could form cell membrane pores and accordingly lead to pyroptosis of retinal microglia to induce damage of the neurovascular unit in DR.

NLRP3 inflammasome signaling is closely related to the maturation of downstream caspase-1^(32)^. Activation of the NLRP3 inflammasome mediates the induction of pyroptosis to further secrete IL-1β^(33)^. Some studies have revealed that the activity of the NLRP3/caspase-1/IL-1β axis was enhanced in microglia located in the central nervous system of patients with Parkinson’s disease^(34-35)^. In the present study, we determined that high glucose could activate NLRP3 inflammasome signaling in retinal microglia, and subsequently trigger pyroptosis. Furthermore, pretreatment with the inhibitors of NLRP3 inflammasome signaling significantly attenuated the high glucose-induced cytotoxicity, activation of the NLRP3 inflammasome, IL-1β secretion, and pyroptosis.

These results confirmed that pyroptosis of retinal microglia was due to high glucose-induced activation of NLRP3 inflammasome signaling. Nevertheless, further studies are warranted to assess whether other signaling pathways are involved in the regulation of pyroptosis in retinal microglia in DR.

In conclusion, treatment with high glucose induced NLRP3 inflammasome-dependent pyroptosis in retinal microglia. This may be one of the main mechanisms resulting in retinal inflammation that initiates or promotes the pathophysiologic progression of DR. Our present findings may provide new potential targets and direction for the therapy of DR.
